# Reducing the Bitterness of Rapeseed Protein: Integrating Enzymatic Treatment, Metabolomics, and Sensory Analysis to Elucidate Underlying Mechanisms

**DOI:** 10.1021/acs.jafc.4c10442

**Published:** 2025-02-02

**Authors:** Andrea Spaccasassi, Christoph Walser, Anni Nisov, Nesli Sozer, Oliver Frank, Corinna Dawid, Thomas F. Hofmann

**Affiliations:** † Chair of Food Chemistry and Molecular and Sensory Science, Technical University of Munich, Lise-Meitner-Str. 34, D-85354 Freising, Germany; ‡ TUM CREATE, 1 CREATE Way, #10-02 CREATE Tower, Singapore 138602, Singapore; § VTT Technical Research Centre of Finland Ltd, Tietotie 2, 02044, Espoo, Finland; ∥ Professorship for Functional Phytometabolomics, TUM School of Life Sciences, Technical University of Munich, Lise-Meitner-Str. 34, 85354 Freising, Germany

**Keywords:** rapeseed/canola, plant-based protein, bitter taste, enzymatic treatment, sensory analysis, metabolomics

## Abstract

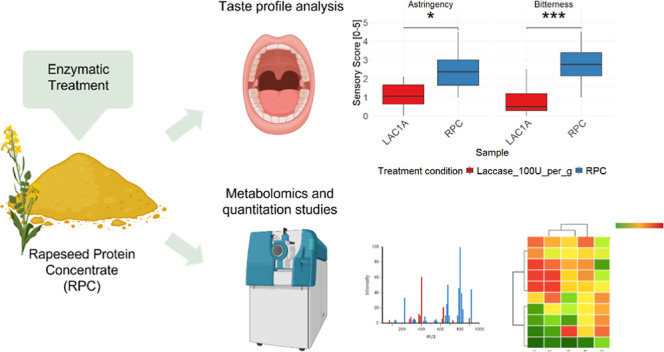

Rapeseed products, such as protein concentrates, hold promise for addressing global protein demands, but their application in food products is limited by their bitter and astringent taste. This study investigates the use of β-glucosidase (BG) and laccase (LAC) enzymatic treatment, individually and combined, to enhance the flavor of rapeseed protein concentrate (RPC). Untargeted metabolomics and sensory analysis reveal that LAC reduces the bitter compound kaempferol 3-*O*-(2‴-*O*-sinapoyl-β-D-sophoroside) (K3OSS) as well as a general reduction in other phenolic compounds, which correlates with a significant decrease in bitterness and astringency. In contrast, BG treatment elevates the levels of K3OSS and is accompanied by increased bitterness due to the conversion of precursor compounds to K3OSS. In addition, the synergistic use of both enzymes significantly reduces the concentration of K3OSS, resulting in a lower perception of bitterness. The LC–MS analysis of pure reference compounds treated with LAC and BG confirms that BG-mediated treatment facilitates the breakdown of larger kaempferol glycosides into K3OSS, while LAC treatment promotes polyphenol polymerization. Consequently, LAC treatment seems to be an effective strategy to improve the sensory quality of RPC and make it more suitable for human consumption.

## Introduction

Due to expected population growth, the global demand for food and, consequently, the demand for proteins is projected to rise significantly.^1^ The consumption of animal-based proteins is associated with environmental concerns, such as high demands for agricultural land, water usage, and greenhouse gas emissions, which makes plant-based proteins increasingly relevant for human nutrition.^2^ Among plant protein sources, canola and rapeseed protein are considered promising supplements to the current protein supply.^3^

Canola protein, derived from *Brassica napus* L. cultivars with lower concentrations of glucosinolates and erucic acid, is recognized for its balanced amino acid composition and techno-functional properties, which make it a valuable food ingredient.^4^ However, its application in the food industry is limited by its undesirable astringency and bitterness, which is primarily attributed to the presence of the key bitter compound kaempferol 3-*O*-(2‴-*O*-sinapoyl-β-D-sophoroside) abbreviated as K3OSS.^5^ Alongside K3OSS, eight other bitter and astringent sensing kaempferol glycosides have been identified in rapeseed protein isolates.^6^ The threshold concentrations at which humans can taste these compounds range from 3.3 to 531.7 μmol/L for bitterness and from 0.3 to 66.4 μmol/L for astringency.^6^

Quantitative analysis using UHPLC-MS/MS-MRM in rapeseed and canola seeds and protein isolates, along with dose-over-threshold (DoT) calculations, indicates a clear impact of several kaempferol glycosides on bitterness and astringency.^6^ Industrial protein production processes further increase K3OSS concentrations by liberating K3OSS from its precursor during processing.^6^

To enhance the palatability of canola protein, strategies such as plant breeding could be used to limit the biosynthesis of K3OSS and its precursors.^7^ In addition, post-production biotechnological modifications, such as fermentation, enzymatic treatments, or solvent extractions, could help to reduce the concentration of off-flavors compounds.^8−11^ Regarding enzymatic treatments, the feruloyl esterase from *Schizophyllum commune* has been shown to reduce K3OSS concentration by 65%, although its effect on bitterness requires further sensory evaluation.^12^ Other enzymes, including β-glucosidases (BGs) and laccases (LACs) like CotA from *Bacillus licheniformis*, may transform K3OSS into less taste-active molecules or alter its stability through oxidation, potentially affecting its sensory properties.^13−15^

This study aims to monitor the enzymatic degradation of K3OSS by determining its concentrations after BG and LAC treatment and characterizing overall metabolomic shifts in rapeseed protein concentrate (RPC) using an untargeted metabolomics approach. By leveraging the information obtained from LC-SWATH-MS and LC–MS/MS analyses, this study also employs sensory evaluation to assess changes in sensory taste profiles. Finally, enzymatic treatments of single and mixed reference compounds are used to validate the results as model studies.

## Materials and Methods

### Chemicals

The following compounds were obtained commercially: formic acid (Merck, Darmstadt, Germany), rutin (Roth, Karlsruhe, Germany), Novozym 51003, sinapinic acid, l-tyrosine, deuterium oxide, and deuterated methanol (Sigma-Aldrich, Steinheim, Germany). The acetonitrile used for HPLC-MS/MS analysis was LC–MS grade (Honeywell, Seelze, Germany). Deionized water for chromatography or enzymatic reactions was purified by using an Advantage A 10 water System (Millipore, Molsheim, France). Bottled water (low mineralization: 405 mg/L, Evian, Wiesbaden, Germany) was adjusted to pH 5.9 with traces of formic acid (0.1% in water) for sensory analysis. Kaempferol 3-*O*-(2‴-*O*-sinapoyl-β-sophoroside)-7-*O*-glucopyranoside and kaempferol 3-*O*-(2‴-*O*-sinapoyl-β-sophoroside) were isolated as reported earlier.^6^

### Raw Material

The main raw material in this study was defatted, milled, and dry fractionated protein-rich (42.2%) RPC. The fraction was produced according to Silventoinen et al. (2022): from a cold-pressed (50–60 °C), pelletized, and air-dried rapeseed press cake from *Brassica rapa* L. seeds (12.9% oil and 30.5% protein) provided by Kankaisten Öljykasvit (Turenki, Finland).^16^ The same methodology was recently applied in another study.^17^ The press cake was precrushed prior to fat extraction by supercritical carbon dioxide (SC–CO_2_), and the fat content was reduced to 2.2%. The defatted press cake was milled twice with a 100 UPZ mill (Hosokawa Alpine AG, Augsburg, Germany) equipped with a 0.3 mm sieve at a rotor speed of 17,800 rpm. The milled material was air-classified with a Minisplit Air Classifier (British Rema, Chesterfield, United Kingdom) at a rotor speed of 4000 rpm, an airflow of 220 m^3^/h, and a feeder speed of 30. The protein-rich rapeseed fraction, that is, the fine fraction gathered by air classification, was used in this study and, in the rest of this paper, it will be referred to as RPC. This RPC was stored at −20 °C.

### Enzymes

The LAC enzyme (Novozym 51003) was commercially obtained, and its activity (90 U/mL of enzyme preparation) was measured at VTT Technical Research Centre of Finland (later referred to as VTT) according to a literature protocol.^14^ The BG enzyme (1140 U activity per mL of enzyme preparation) was produced at VTT.^18^

### Enzymatic Treatment

The RPC was treated with LAC, BG, and a combination of these two enzymes at varying temperatures (50 °C and room temperature [RT]) and pH values (6.1 and 5). The treatments consisted of 6 U/g RPC LAC and 100 U/g RPC BG (Table 1). RPC suspensions (10% w/v) were prepared and maintained at the desired temperature with constant stirring. The pH was either left at its natural value (6.1) or adjusted to 5 prior to enzyme addition. Enzyme treatments included LAC (6 U/g RPC), BG (100 U/g RPC), or both enzymes, incubated for 2 h. A sequential treatment was also tested, where LAC treatment (RT, 2 h) was followed by BG treatment (50 °C, 2 h). Control samples received deionized water instead of enzymes to maintain volume consistency. After incubation, pH was readjusted to 6.1 using HCl (6 mol/L) or NaOH (6 mol/L). Selected samples were inactivated at 90 °C for 10 min, cooled in an ice bath, and frozen at −20 °C; non-inactivated samples were directly frozen. All samples were freeze-dried. Samples treated with LAC (6 U/g RPC) were coded “LAC1” (rep A and B, pH 6.1, dosage 6 U/g RPC, RT with inactivation step 90 °C × 10 min), while control LAC samples were coded “LAC2” (rep A and B, inactivated at 90 °C × 10 min) and “LAC3” (rep A and B where no inactivation was performed). Samples treated with both “LAC” and “BG” were coded as “LB1” (rep A and B, treated first with LAC for 2 h at RT followed by treatment with BG at 50 °C for 2 h) and “LB2” (rep A and B, treated with LAC and BG (LB) together at RT for 2 h). Samples treated with BG were coded as “BG2” (pH 5, 100 U/g RPC, 50 °C, 2 h, with inactivation), “BG3” (pH 6.1, 100 U/g RPC, 50 °C, 2 h, with inactivation), “BGL 1 no in” (pH 5, 100 U/g RPC, 50 °C, 2 h, without inactivation), and “BGL2 no in” (pH 6.1, 100 U/g RPC, 50 °C, 2 h, without inactivation). The control samples for BG treatment were coded “BG1” (pH 5, 0 U/g RPC, 50 °C, 2 h, with inactivation), “BG3 no in” (pH 5, 0 U/g RPC, 50 °C, 2 h, without inactivation), “BGL10” and “BGL 11” (pH 6.1, 0 U/g RPC, 50 °C, 2 h, without inactivation), and “BGL 12” (pH 5. 0 U/g RPC, 50 °C, 2 h, without inactivation). The sample codes and related treatments are summarized in Table 1.

**Table 1:** Table Summary of the Enzyme Treatments

sample code^a^	enzyme^b^	pH	dosage (U/g RPC)	treatment^c^	inactivation^d^
BG1	BG	5	0	50 °C, 2 h	90 °C × 10 min
BG2	BG	5	100	50 °C, 2 h	90 °C × 10 min
BG3	BG	6.1	100	50 °C, 2 h	90 °C × 10 min
BGL 1 no in	BG	5	100	50 °C, 2 h	no
BGL 2 no in	BG	6.1	100	50 °C, 2 h	no
BG3 no in	BG	5	0	50 °C, 2 h	no
BGL10	BG	6.1	0	50 °C, 2 h	no
BGL11	BG	6.1	0	50 °C, 2 h	no
BGL12	BG	5	0	50 °C, 2 h	no
LAC1	LAC	6.1	6	RT	90 °C × 10 min
LAC2	LAC	6.1	0	RT	90 °C × 10 min
LAC3	LAC	6.1	0	RT	no
LB1	LAC + BG	6.1	LAC:6 BG:100	RT, 2 h + 50 °C, 2 h	90 °C × 10 min
LB2	LAC + BG	6.1	LAC:6 BG:100	50 °C, 2 h	90 °C × 10 min
RPC	LAC + BG	5	0	50 °C, 2 h	no

a RPC represents the untreated rapeseed protein concentrate.

b BG denotes β-glucosidase. LAC denotes laccase. LAC + BG denotes the combined treatment.

c RT denotes room temperature.

d When inactivation = no, the enzyme inactivation step (90 °C × 10 min) was not performed.

### Enzymatic Treatment of Pure Reference Compounds

To assess the impact of enzymatic treatments with BG and LAC on kaempferol metabolites, reference compounds obtained from the study of Walser et al. (2024),^6^ kaempferol 3-*O*-(2‴-*O*-sinapoyl-β-sophoroside) (**1**, K3OSS), and kaempferol 3-*O*-(2‴-*O*-sinapoyl-β-sophoroside)-7-*O*-glucopyranoside (K3OSS7OG) were subjected to various treatments either individually or in combination. LAC (RT, 2 h) and BG (50 °C, 2 h) treatments as well as their combination treatments (50 °C, 2 h) were performed under the conditions described in the previous section, including enzymatic treatments, inactivation, and freeze-drying. As the target was to expose the pure components to maximal enzyme treatment, the compound solutions were overdosed with the enzymes. Stock solutions of LB enzyme dilutions were prepared and dosed according to Table 2. The samples were prepared as follows: each compound (sinapic acid, K3OSS, and K3OSS7OG) was treated with BG, LAC, or a combination of both (LB) under different temperature conditions and enzyme dosages. The samples containing BG were incubated at 50 °C, and those containing LAC were treated at RT. For the combined enzyme treatment (LB), both enzymes were used in equal doses of 500 units each and treated at 50 °C. The treatments were arranged in the following manner. In the BG treatments (samples s1, s7, s10, s13, and s16), the compounds were treated with BG at 50 °C using a dose of 1000 units of BG. Each compound was incubated separately to obtain samples s1 (sinapic acid, K3OSS, and K3OSS7OG), s7 (sinapic acid), s10 (K3OSS), s13 (K3OSS7OG), and s16 (control). The LAC treatments (samples s2, s8, s11, s14, and s17) were performed at RT with a dose of 1000 units of LAC. The individual compounds were treated separately to produce samples s2 (sinapic acid, K3OSS, and K3OSS7OG), s8 (sinapic acid), s11 (K3OSS), s14 (K3OSS7OG), and s17 (control).

**Table 2:** Summary of the Enzymatic Treatment of Reference Compounds

sample code	compounds^a^	enzyme treatment^b^	temperature^c^	dose LAC (U/g RPC)	dose BG (U/g RPC)
**s1**	sinapic acid K3OSS, K3OSS7OG	BG	50	0	1000
**s2**	sinapic acid K3OSS, K3OSS7OG	LAC	RT	1000	0
**s3**	sinapic acid K3OSS, K3OSS7OG	LB	50	500	500
**s7**	sinapic acid	BG	50	0	1000
**s8**	sinapic acid	LAC	RT	1000	0
**s9**	sinapic acid	LB	50	500	500
**s10**	K3OSS	BG	50	0	1000
**s11**	K3OSS	LAC	RT	1000	0
**s12**	K3OSS	LB	50	500	500
**s13**	K3OSS7OG	BG	50	0	1000
**s14**	K3OSS7OG	LAC	RT	1000	0
**s15**	K3OSS7OG	LB	50	500	500
**s16**	zero BGL	BG	50	0	1000
**s17**	zero LAC	LAC	RT	1000	0
**s18**	zero LB	LB	50	500	500

a K3OSSS = kaempferol 3-*O*-(2‴-*O*-sinapoyl-β-d-sophoroside) and K3OSS7OG = kaempferol 3-*O*-(2‴-*O*-sinapoyl-β-d-sophoroside)-7-*O*-β-d-glucopyranoside. Zero LB, zero LAC, and zero BGL are the control samples (enzymes only) in which compounds were not included.

b BG denotes β-glucosidase, LAC denotes laccase, and LB denotes the combination of LAB and BG.

c RT denotes room temperature.

In the combined treatment (LB; samples s3, s9, s12, s15, and s18), both LAC and BG were used in equal doses of 500 units each at 50 °C. The compounds were treated individually to obtain samples s3 (sinapic acid, K3OSS, and K3OSS7OG), s9 (sinapic acid), s12 (K3OSS), s15 (K3OSS7OG), and s18 (control). The obtained samples were then analyzed using the untargeted metabolomics method described below.

### Sensory Analysis

#### Sensory Panel Training and Sample Pretreatment

The sensory panel contained 22 panelists (11 females and 11 males, 23–35 years in age) who had given informed consent to perform sensory tests. These panelists had been trained weekly with reference taste compounds for at least one year to allow them to become familiar with the sensory methodologies used in this study and to evaluate different chemosensory qualities.^19^ The sensory analyses were performed in sensory cabins at 22–25 °C using nose clips under yellow light to avoid cross-model interactions with odorants and colors.

#### Comparative Profile Sensory Analysis

A portion (1.5 g) of the samples coded as BGL2B, RPC, LB2A, LAC1A, LAC2A, LB2A, and LB1B (Table 1) were suspended in Evian water (25 mL, pH 5.9) and, after centrifugation, presented to the trained panelists together with the same amount of an untreated RPC sample as a reference. The panel was asked to evaluate the bitter, astringent, sour, sweet, umami, and licorice-like taste perceptions on a scale from 0 (not detectable) to 5 (strongly detectable) in comparison to the untreated RPC.

### Sample Preparation for Analytical Analysis

The samples (200 mg ± 10 mg each, cf. Table S1) were weighted into Precellys homogenizing tubes (Lysing Kit, Dry Hard Tissue Grinding, 15 mL, Bertin Technologies, Montigny-le-Bretonneux, France) in duplicates. Solvent (5 mL, MeOH/H2O, 8/2, v/v) and internal standard (IS = rutin, 10 μL (*c* = 52.25 mg/L)) were added to each sample, and the tubes were cooled at −20 °C for 2 h. The samples were homogenized using a Precellys Evolution homogenizer (Bertin Technologies, Montigny-le-Bretonneux, France) at 5000 rpm at intervals of 30 s with breaks of 30 s between the intervals. The homogenized samples were centrifuged (Eppendorf centrifuge 5810 R, Hamburg, Germany) at 4000 rpm for 15 min at 10 °C. The supernatant was filtered with a membrane filter (Minisart RC, 0.45 μm, Sartorius AG, Goettingen, Germany) directly into vials for liquid chromatography–mass spectrometry analysis (LC–MS). Furthermore, a pooled sample containing equal amounts of each other sample was prepared. This sample (200 mg ± 10 mg) was weighed and submitted to the same sample workup described above to obtain a quality control sample (QC).

### Quantitation of the Kaempferol Glycosides

The quantitation was performed according to Walser et al. (2024).^6^ An AB Sciex 5500 Qtrap mass spectrometer (Sciex, Darmstadt, Germany) with direct flow infusion was used to acquire the mass and product ion spectra. The instrument was controlled with the Analyst software (version 1.6.2, Applied Biosystems, Darmstadt, Germany) and operated in full-scan mode (negative, ion spray voltage, −4500 V): curtain gas at 35 psi, a temperature of 400 °C, gas 1 at 45 psi, gas 2 at 65 psi, a collision-activated dissociation medium, and an entrance potential of −10 V. Compounds **1**–**9** and the IS were dissolved in acetonitrile and water and infused to give the specific product ions and ionization parameters as described.^3^

The MS system was connected with a Shimadzu Nexera X2 UHPLC system (Sciex, Darmstadt, Germany) consisting of a DGU-20A 5R degasser, two LC30AD pumps, a SIL30AC autosampler (kept at 15 °C), and a CTO30A column oven (40 °C). Separation of the compounds was performed on a Kinetex C18 column (100 mm × 2.1 mm, 1.7 μm, 100 Å, Phenomenex, Aschaffenburg, Germany) by injecting aliquots (2 μL) of the samples into the system running at a flow rate of 0.4 mL/min. Water and acetonitrile, each with formic acid (0.1%), were used as solvents A and B. The following gradient was used for separation: initial conditions, 5% B, maintained at 5% for 3 min, increased within 2 min to 15% B, increased in 4 min to 30% B, increased in 1 min to 100% B, maintained at 100% B for 2 min, decreased over 1 min to 5% B, and held for 2 min isocratically.

### UPLC/Time-of-Flight Mass Spectrometry

UPLC/time-of-flight mass spectrometry (UHPLC/ToF–MS) measurements were performed on a Sciex TripleTOF 6600 mass spectrometer (Sciex, Darmstadt, Germany) connected to a Shimadzu Nexera X2 system (Shimadzu, Duisburg, Germany) operating in negative electrospray ionization (ESI) mode. Instrumentation control and data acquisition were performed with AnalystTF software (version 1.7.1, Sciex, Darmstadt, Germany). The ion spray voltage was set at −4500 eV, the source temperature was set at 550 °C, the nebulizing gas was set at 55 psi, and the heating gas was set at 65 psi. Nitrogen served as the curtain gas at 35 psi to effectively desolvate the ions. Chromatography was performed on a Kinetex C18 column (150 × 2 mm, 1.7 μm, Phenomenex, Aschaffenburg, Germany) equipped with a guard column. The gradient consisted of solvent A [aqueous formic acid (0.1%)] and solvent B [acetonitrile containing formic acid (0.1%)] at a flow rate of 0.3 mL/min. The following gradient was used for separation: 0 min, 5% B; 2 min, 5% B; 18 min, 100% B; 19 min, 100% B; 20 min, 5% B; 17 min, 5% B; and 22 min, 5% B. The column oven temperature was set at 40 °C, and the injection volume was 5 μL. The samples used for the quantitative methodology were also used for the untargeted analysis.

#### Data-Independent Acquisition

In SWATH mode, ToF–MS survey scans were acquired (*m*/*z* 50–1,500, 100 ms), followed by 23 product ion scans with variable Q1 isolation windows from *m*/*z* 50 to 1349 with an overlap of 1 Da: *m*/*z* 50–72.8, 71.8–113.0, 112.0–144.8, 143.8–174.4, 173.4–202.4, 201.4–229.1, 228.1–258.4, 257.4–288.4, 287.4–321.9, 320.9–357.4, 356.4–397.7, 396.7–437.7, 436.7–467.5, 466.5–497.8, 496.8–528.3, 527.3–568.0, 567.0–622.5, 621.5–678.6, 677.6–755.4, 754.4–903.4, 902.4–1050.0, 1049.0–1150.0, and 1149.0–1349. Product ion spectra were accumulated in the high-resolution mode for 50 ms using a declustering potential (DP) of 80 V, a collision energy (CE) of −35 V, and a collision energy spread (CES) of 15 V. System control and data acquisition were performed by using AnalystTF (version 1.7.1, Sciex, Darmstadt, Germany).

### Data Analysis, Visualization, and Statistical Evaluation

Acquired data were preprocessed using MS-DIAL software (version 5.2.230912).^20^ Peak picking was performed with MS1 and MS2 tolerances set to 0.1 Da, a minimum peak height of 1000, and a mass slice width of 0.1 Da. Linear weighted average smoothing (level 4) and a minimum peak width of five scans were applied. QC samples, prepared as a mixture of all samples and processed identically, were injected at regular intervals and used as references for alignment. The alignment table was filtered to exclude ions present in blanks and exported from MS-DIAL for further processing in R (version 4.1.2) using ggplot2, ggpubr, and ComplexHeatmap.^21,22^ Multivariate statistical analysis was performed using the principal component analysis (PCA) function from the FactoMineR package and visualized using the ggplot2 package.

Metabolite selection from the untargeted metabolomics data set was based on querying the peak master from the alignment table and MS/MS spectra SWATH fragmentation data, focusing on taste-active compound fingerprints. MS/MS spectra were filtered for a signal-to-noise ratio ≥500, containing specific fragments (*m*/*z* 284, 255, 771, 609, 446, 429, 815, 623, 977, 591, and 1183) selected based on known metabolite fragmentation patterns and identified spectra containing at least two such masses.^6^ Further filtering was applied to these spectra based on adduct type ([M – H]^−^) and retention time (2 to 9 min). The alignment IDs from spectra meeting these criteria were used to select the corresponding features from the peak table. These features were annotated with a unique identifier combining average mass-to-charge ratio (*m*/*z*) and retention time (*t*_r_). Selected alignment spots were then annotated using MSFINDER software (version 3.60) to determine formula and structure annotation.^23,24^

Sensory analysis results were plotted in box plots using the ggplot2 package, and mean comparisons were obtained using the function “stat_compare_means” from the ggpubr.

## Results and Discussion

### Sensory Analysis

The untreated RPC exhibited sensory scores of 2.5 for astringency, 2.8 for bitterness, 0.5 for burning, 0.3 for licorice, 0.1 for saltiness, 0.9 for sourness, 0.6 for sweetness, and 0 for umami. These values indicate that bitterness and astringency are the most prominent sensory attributes. Enzymatic treatments were employed to address the negative impact of these attributes on the rapeseed protein. Taste profile analysis of the treated samples (BGL2B, LB2A, LAC1A, LAC2A, LB2A, and LB1B; cf. Table 1) revealed that the enzymatic treatments had significant influences on the taste profiles, suggesting their potential for improving the sensory characteristics of rapeseed protein. Specifically, bitterness perceptions of samples LAC1, LB1, and LB2, which were treated with LAC or a combination of LAC and BG, demonstrated statistically significant (*p* < 0.05) reductions compared to the untreated RPC sample and non-LAC-treated samples such as LAC2 and BGL2 (Figure 2). The average bitter sensory score decreased from 2.8 (RPC) to 0.8 for LAC1A, 1.2 for LB1B, and 0.7 for LB2B. Conversely, samples LAC2 and BGL2, representing a LAC control and a BG-treated sample, respectively, did not exhibit significant changes in bitterness levels (*p* > 0.05). A comparable trend was observed in perceived astringency, with samples LAC1, LB1, and LB2 showing substantially lower average astringency scores (*p* < 0.05) than the RPC (Figure 2). Astringency scores decreased from 2.5 (RPC) to 1.1 for LAC1, 1.5 for LB1, and 1.1 for LB2. Attributes such as the burning sensation, licorice taste, saltiness, sourness, and umami were unaffected (*p* > 0.05). Notably, sample LAC1 was recognizably sweeter than the control, suggesting that LAC-mediated debittering and subsequent K3OSS degradation might enhance sweetness perceptions. In summary, the data indicate that LAC treatment positively modifies the sensory profile of the RPC. In contrast, BG treatment seemed to increase bitter off-tastes (from 2.8 to a 3.3 average score), although this difference was not statistically significant (*p* > 0.05). Overall, these results are very promising in indicating that enzymatic treatment can improve the taste of rapeseed protein and, thus, make it more suitable for human consumption. In the following section, investigations are carried out at the molecular level and linked to the sensory results.

### Untargeted Metabolomics Mapping

Given the sensory improvements observed in the enzymatically treated RPCs, an untargeted metabolomics approach was employed to comprehensively evaluate the biochemical changes that occurred during these treatments. The goal was to investigate the shifts in the metabolomic profile, particularly focusing on the transformation of key compounds linked to bitterness and astringency. From the analysis of negative ESI mode LC-SWATH-MS data, a total of 32,488 features were detected. The known bitter compound **1** (K3OSS) was attributed to a feature with a retention time of 6.29 min and a pseudomolecular ion ([M–H]^−^) at *m*/*z* 815.2023 (referred to as 815.2023_6.29). MS/MS fragmentation in the negative ESI mode showed the fragment ions at *m*/*z* 609 [M–H–sinapoyl]^−^, 591 [M–H–sinapoyl–H_2_O]^−^, 429 [M–H–sinapoyl–H_2_O–Glc]^−^, and 284 [M–H–sinapoyl–2 Glc/kaempferol–2H]^−^, as reported previously.^5^ The pure reference compound from Hald et al. (2019) was used to confirm the identity of K3OSS.^5^ The retention time and fragmentation pattern were consistent with those of the pure reference compound. The full alignment file was subsequently summarized by using PCA to examine potential metabolome changes following enzymatic treatment. The biplot generated from this analysis is shown in Figure 3A. The figure shows a significant shift in metabolome profile when samples are treated with LAC, regardless of whether this was done in combination with BG or alone. The control samples LAC2 and LAC3 showed a significant difference in distance compared to the treated samples LAC1, LB1, and LB2 and, therefore, suggest that major metabolome changes occurred during treatment with LAC or a combination of LAC and BG.

**Figure 1:**
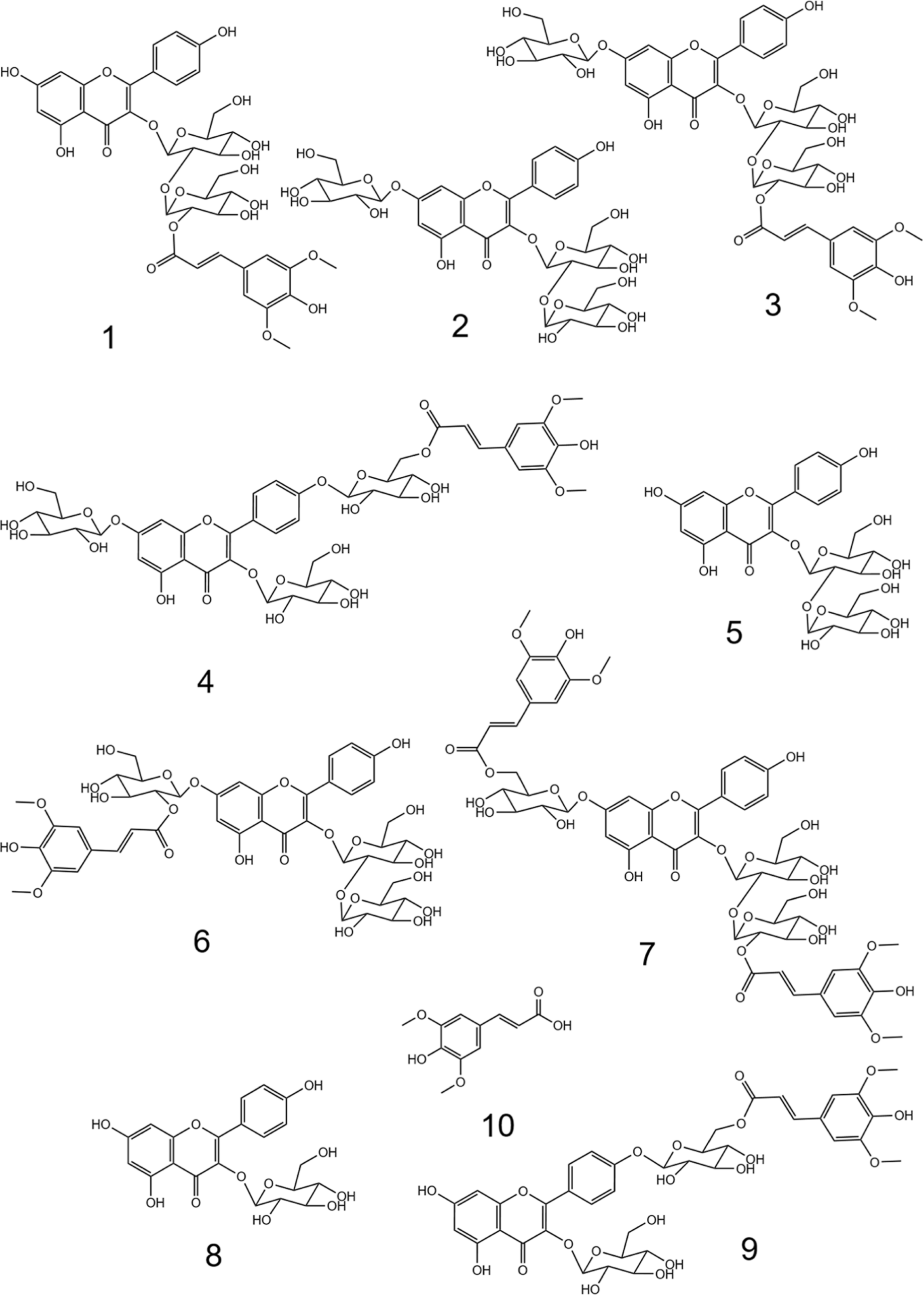
Chemical structures of bitter tasting kaempferol glycosides and sinapic acid: kaempferol 3-*O*-(2‴-*O*-sinapoyl-β-D-sophoroside) (**1**), kaempferol 3-*O*-β-D-sophoroside-7-*O*-β-d-glucopyranoside (**2**), kaempferol 3-*O*-(2‴-*O*-sinapoyl-β-D-sophoroside)-7-*O*-β-d-glucopyranoside (**3**), kaempferol 4′-*O*-(6-*O*-sinapoyl-β-d-glucopyranoside)-3,7-di-*O*-β-d-glucopyranoside (**4**), kaempferol 3-*O*-β-D-sophoroside (**5**), kaempferol 3-*O*-β-D-sophoroside-7-*O*-(2-*O*-sinapoyl-β-d-glucopyranoside) (**6**), kaempferol 3-*O*-(2‴-*O*-sinapoyl-β-d-sophoroside)-7-*O*-(6-*O*-sinapoyl-β-d-glucopyranoside) (**7**), kaempferol 3-*O*-β-d-glucopyranoside (**8**), kaempferol 4′-*O*-(6-*O*-sinapoyl-β-d-glucopyranoside)-3-*O*-β-d-glucopyranoside (**9**), and sinapic acid (**10**).

**Figure 2:**
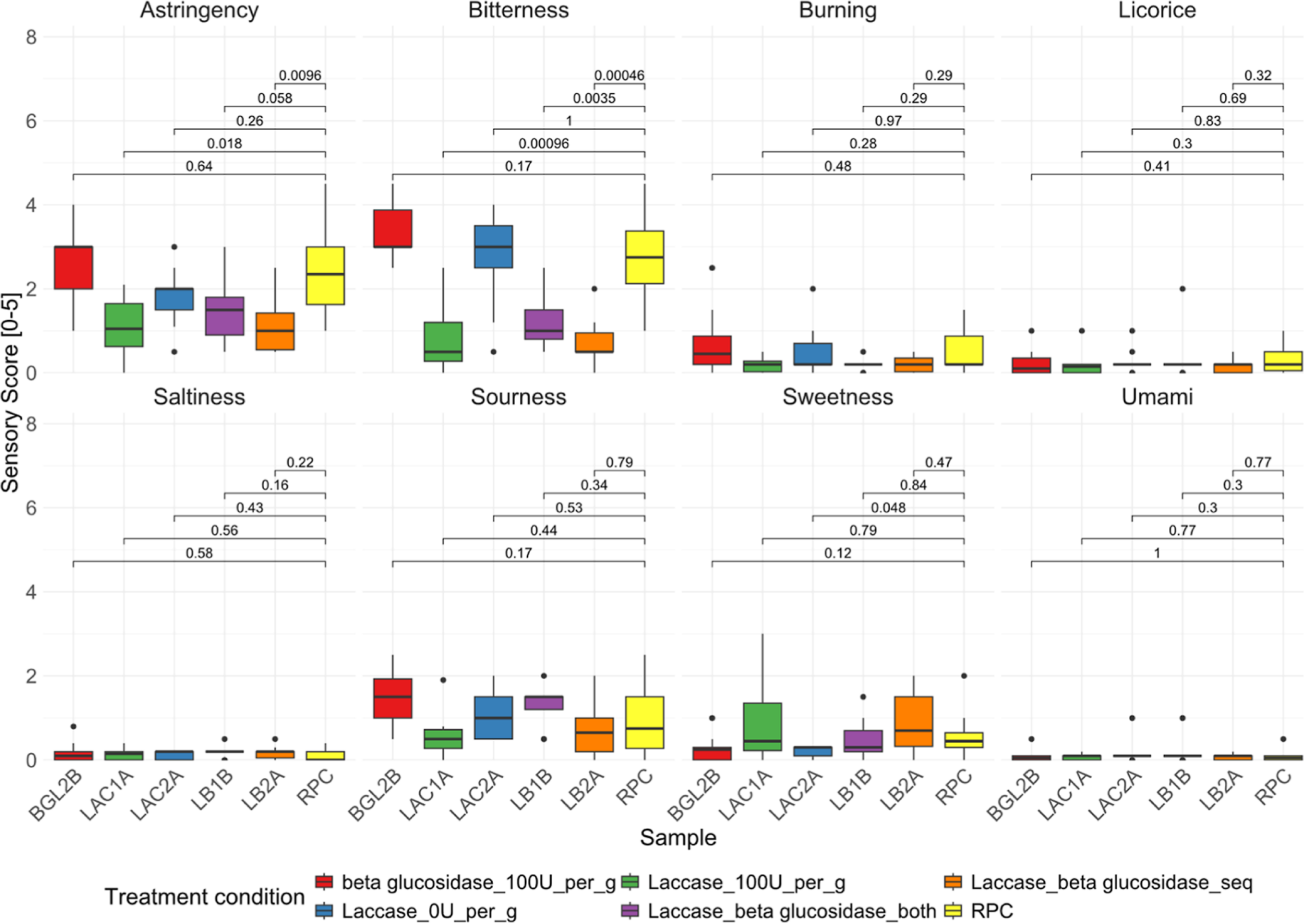
Human sensory profile analysis of the RPC treated with the enzymes BG and LAC and controls. Samples included are BGL2 (treated with BG), LAC1 (treated with LAC), LAC control (LAC2), LB1 (treated with LAC and BG together), LB2 (treated with LAC and BG sequentially), and the untreated RPC. Each plot facet shows the measured sensory attributes, whereas each colored boxplot shows the distribution of the sensory score for each sample for that particular attribute. Statistical significance is computed and reported in the graph.

**Figure 3:**
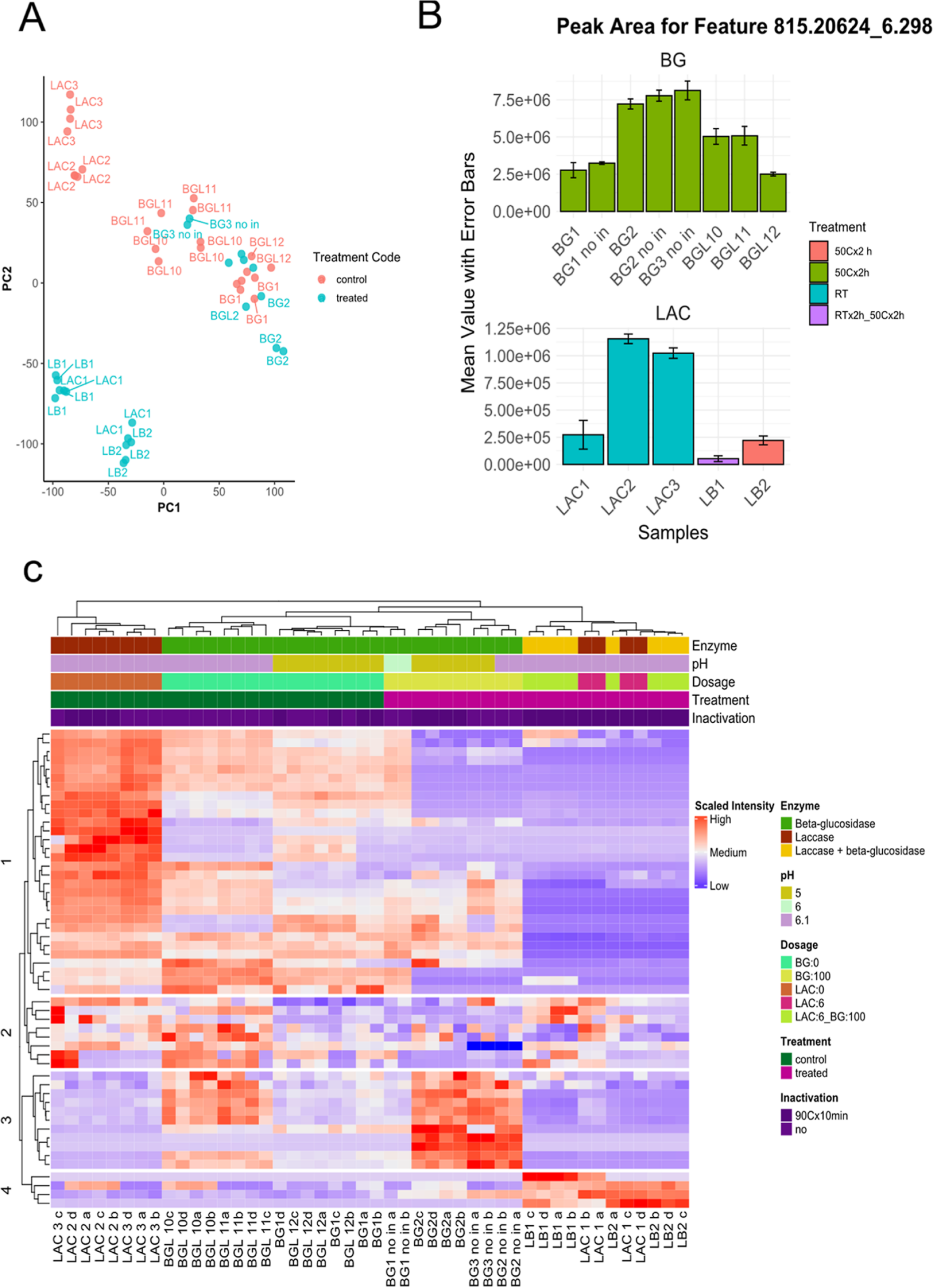
Multipanel graphical representation of the untargeted metabolomics profiling of the enzymatically treated samples. Panel A is an individual plot obtained from the PCA of the full alignment file. Panel B is a bar plot showing the mean peak area of feature 815.20624_6.298 across different samples. The bars represent the mean values, while the error bars indicate the standard deviation for each sample within the respective treatment groups. Samples are grouped by the enzyme treatment and presented in different facets to allow for independent y-scales. Panel C is a heatmap of the selected feature from the alignment file, where target fragment masses were identified. Color is based on the scaled peak area extracted from the peak table. A larger version of panel C which includes the features’ names can be found in the Supporting Information (Figure S1).

Additionally, samples treated with BG also showed a shift, albeit to a lesser degree than that of samples treated with LAC. The results indicate that LAC treatment significantly affected the metabolome of the rapeseed protein. By comparing the peak area of feature 815.2023_6.29 (compound 1, Figure 1) in the bar plot shown in Figure 3B, it is evident that LAC, whether used alone or in conjunction with BG, significantly decreased the concentration of 815.2064_6.29. Samples LAC1A and LAC1B treated with LAC showed a very low intensity of this feature. Samples LB1A and LB1B, which were treated with BG and LAC in two different steps, showed the most noticeable decrease in intensity (Figure 3B). Samples LB2A and LB2B, when treated with a mixture of LAC and BG in a single step (Table 1), also exhibited a decrease in intensity, even though it was lower than that of the LB1 samples (Figure 3B). In contrast, the control samples (LAC2 and LAC3) did not show the same reduction as their treated counterparts, although a decrease in peak area was observed compared to that of the BG control samples (Figure 3B). However, this reduction did not affect the sensory outcomes (Figure 2). A possible reason behind this reduction could be the incubation of the samples at RT. This might have activated endogenous enzymatic transformation or fermentation processes, thereby affecting the intensity of the specific compound cluster (Figure 3C and Figure S1 row k-mean cluster 3). Control samples of BG, which were incubated at 50 °C, did not show this degradation (Figure 3C and Figure S1, row k, mean cluster 3). Major compound clusters in the LAC control samples LAC2 and LAC3 (Figure 3C, row k-mean cluster 1) also seemed unaffected, which suggests that no analytical error or sample preparation issue caused the reduction in row k-mean cluster 3 where 815.2064_6.29 is located. The inactivation step at 90 °C × 10 min or incubation at pH 5 did not seem to impact the control samples BG1, BG11, BG12, and “BGL 3 no in”, since they did not show a decrease in peak area. The only variable associated with a decrease in peak area is the incubation of 10% RPC in a water suspension at RT.

Interestingly, samples BGL2B and BGL3B, which were treated with BG at pH levels of 5 (adjusted) and 6.1 (native), showed an increased intensity of **1** (Figure 3B). Regardless of the pH value, this increase implies that **1** was not degraded by treatment with BG but was increased due to the possible transformation of a precursor metabolite into **1**. To investigate these changes in more detail, the alignment file containing information on the SWATH-acquired MS/MS spectra was searched for known kaempferol glycoside fragments to select features according to their known fragment masses.^6^ Among the features containing relevant fragments, several potential precursor ions were selected: the feature with *m*/*z* 1183.3158 [M – H]^−^ at a retention time of 6.85 min with fragment ions at *m*/*z* 815, 609, 429, and 285, which was previously reported as kaempferol 3-*O*-(2‴-*O*-sinapoyl-β-d-sophoroside)-7-*O*-(6-*O*-sinapoyl-β-d-glucopyranoside) (**7**); the feature with *m*/*z* 977.25604 [M – H]^−^ at a retention time of 5.28 min with fragment ions at 815, 609, and 284, which was previously identified as kaempferol 3-*O*-β-d-sophoroside-7-*O*-(2-*O*-sinapoyl-β-d-glucopyranoside) (K3OS7SG, **6**); and the feature with *m*/*z* 977.25691 at a retention time of 6.3 min and fragment ions at 815, 609, and 184, identified as kaempferol 3-*O*-β-d-sophoroside-7-*O*-(2-*O*-sinapoyl-β-d-glucopyranoside). These features showed a drastic reduction in intensity following treatment with LAC, similar to compound **1**. In contrast, treatment with BG led to their complete degradation (Figures S2–S4 and Figure 3C, row k, mean cluster 1). This result confirms the activity of LAC treatment against kaempferol derivatives and suggests that BG can catalyze the cleavage of sugar in an external position such as the 7-*O*-glucoside moiety (compound **3**, Figure 1). However, this effect is not evident in the case of bitter compound **1** because of the absence of a glycosidic bond in the external position. In addition to **1**, the absence of a glycosidic bond in the external position for the feature 1183.3165 [M – H]^−^ at a retention time of 6.69 min (**7**, Figure 1) was unaffected by BG but strongly influenced by LAC (Figure S5). This missing cleavage is possibly related to the steric hindrance of the kaempferol and sinapic acid moieties, which prevents the BG enzyme from catalyzing the glycosidic bond hydrolysis. Neighboring residues in the active site pocket of BG produce steric hindrance. This might imply that BG kinetics rely on substrate configuration.^25^ This hypothesis is further supported by observing the feature with *m*/*z* 815.2074 [M – H]^−^ at a retention time of 6.99 min with fragment ions at 653, 447, and 285, which was tentatively identified as kaempferol 4′-*O*-(6-*O*-sinapoyl-β-d-glucopyranoside)-3-*O*-β-d-glucopyranoside (**9**). In contrast to compound **1**, this compound has a glycosidic bond at position 7, allowing BG to access the cleaving point more freely, thereby catalyzing the hydrolysis. Indeed, a clear reduction in intensity was observed for samples treated with BG (Figure S6). Furthermore, the BG-catalyzed hydrolysis of precursor **3** likely results in the formation of an additional amount of **1**. This pattern was evident in the samples BGL2B and BGL3B, which showed larger peak area intensities for **1** (815.2064_6.29) after BG treatment (Figure 3B and Figure S3). Therefore, the increased perceived bitterness in the sample BGL2B, as shown in Figure 2, can be directly attributed to this mechanism. By extracting alignment features with patterns indicating the activity of the LAC enzyme, it is possible to observe the overall effect of the LAC enzyme on kaempferol and sinapic acid derivatives. A metabolomics feature heatmap is presented in Figure 3C. Among the metabolites profoundly reduced in intensity upon LAC treatment, a feature with *m*/*z* 223.0604 [M – H]^−^ at a retention time of 6.37 min was identified as sinapic acid. Additionally, a feature with *m*/*z* 609.1409 [M – H]^−^ at a retention time of 6.04 min, with fragment ions at 609 and 284, was identified as kaempferol 3-*O*-β-D-sophoroside (K3OS, **5**). Another feature, with *m*/*z* 447.0906 at a retention time of 6.68 min and fragment ions at 447 and 284, was identified as kaempferol 3-*O*-β-d-glucopyranoside (K3OG, **8**).

In conclusion, LAC treatment significantly affects all phenolic-containing molecules regardless of their specific structure, whereas BG treatment primarily targets metabolites with sugar moieties in external positions. BG treatment results in the degradation of certain precursor compounds and the formation of smaller kaempferol derivatives. Notably, the pH and the inactivation step do not influence the outcomes of BG or LAC treatment. The degradation of the bitter compound **1** appears to be driven by LAC treatment, whether applied alone or in combination with BG, although slight differences can be observed. The incubation of control samples LAC2 and LAC3 seems to have an impact, even though the reasons for this are difficult to pinpoint with the available literature and data. Nevertheless, all of these findings provide a deeper understanding of the molecular changes occurring during treatment and help to explain the sensory changes depicted in Figure 2. In the following section, quantitative data are presented to further explain the results of the enzymatic treatment.

### Targeted Quantification of the Bitter-Tasting Kaempferols

**Figure 4:**
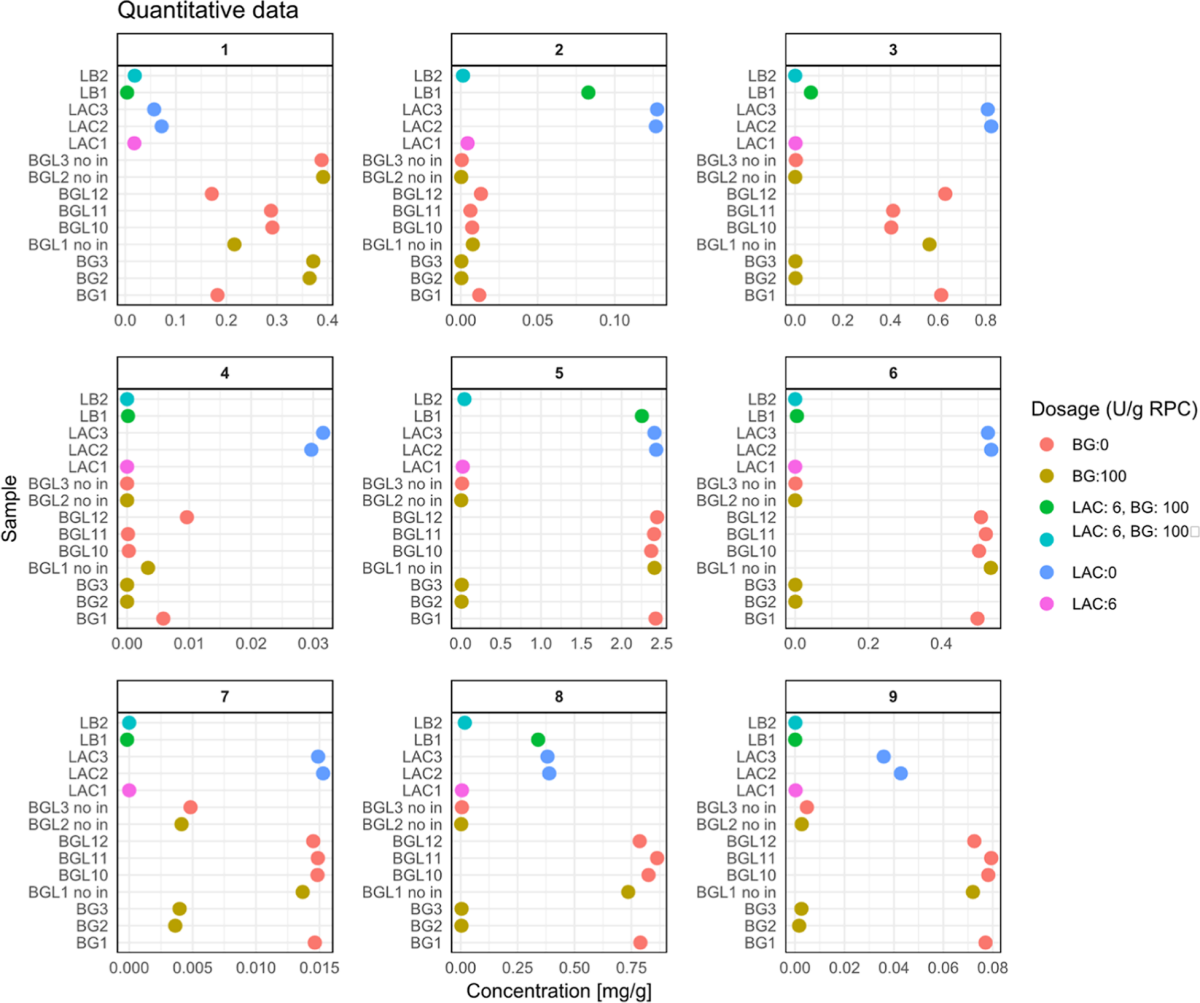
Scatter plot matrix showing the quantitative analysis of kaempferol at varying concentrations across different sample groups. Each plot compares the concentrations (mg/g) of kaempferol in the samples, measured in response to various enzyme treatments. Sample groups are distinguished by color coding.

To obtain a quantitative insight and confirm the untargeted metabolomics results, the known bitter and astringent-tasting kaempferol glycosides were quantified in the samples according to Walser et al. (2024).^6^ Quantitative data for the nine taste-active kaempferols are graphically represented in Figure 4 and detailed in Table S1. Confirming the untargeted results, the quantitative data indicate a drastic drop in the concentration of bitter compound **1** in the samples treated with laccase LAC1, LB1, and LB2 (down to 0.018, 0.003, and 0.019 mg/g). This reduced concentration is well in line with a significant reduction in bitterness and confirms the sensory analysis results (Figure 2).

Furthermore, samples treated with BG, such as BGL3 and BGL2, showed an increase in concentration for **1** (0.36 and 0.37 mg/g) compared to that of the untreated samples BGL1, BGL10, BGL11, and BGL12 (0.19–0.30 mg/g). This observation is consistent with the nontargeted results indicating the conversion of precursor metabolites to bitter kaempferol derivatives during treatment with BG due to the cleavage of glycosidic bonds in the external position. The increased concentration of bitter compound **1** is reflected by the observed sensory results, although statistical significance was not obtained. The quantitative data also showed a decrease in concentration for samples LAC2 and LAC3. However, according to the sensory data, this effect was not significant, since the LAC2 sample was judged to be as bitter as the untreated sample (Figure 2). As reported in the untargeted section, the only variable associated with this decrease was the incubation at RT that LAC2 and LAC3 underwent, which did not occur for the BG controls (incubation at 50 °C). This incubation time might result in degradation processes, but the available literature is too limited to be able to pinpoint a possible mechanism for this effect.

Interestingly, just as in the untargeted results, the concentration of compounds **2**, **3**, **4**, **6**, and **7** appeared to decrease during treatment with both LAC and BG. This observation confirms our hypothesis that BG can only cleave glycosidic bonds when they are at external positions, such as positions 3 and 7 for compounds **2**, **3**, **4**, **5**, **6**, **8**, and **9**. In contrast, compounds **1** and **7** do not have glycosidic bonds at the external position and do not appear to be degraded by BG (Figure 4). The degradation of compound **3** with BG might be linked to the increase in the concentration observed for compound **1**. Compound **5** presents a significant degradation and reduction in concentration during treatment. This compound’s concentration is in the 2.4 mg/g range in the untreated samples and goes down to the 0.01–0.04 mg/g range upon treatment with BG, LAC, or BG and LAC combined, independently from the pH or inactivation. This effect was also shown for compound **8**, which appears to be reduced in a similar pattern, ranging between 0.8 and 0.3 mg/g down to 0.01–0.002 mg/g in the treated samples. The efficacy of the LAC-treated sample on compounds **5** and **8** (Figure 4) confirms that LAC-mediated polyphenol oxidation was occurring on the kaempferol side as well and not exclusively on the sinapic acid moiety. Next, model studies will be used to confirm the results obtained so far and to obtain further insights into the enzymatic treatment of kaempferol derivatives and sinapic acid.

### Treatment of Single Reference Compounds

To prove, in a simplified model, the enzyme effect observed in the untargeted metabolomics and quantitative results, enzymatic treatments of individual pure reference compounds were performed. Solutions of the reference compounds were treated with LB enzymes. The compounds **1** (K3OSS), **3** (K3OSS7OG), and **10** (sinapic acid) were included and enzymatically treated, both individually and in combination. Samples obtained from this experiment were screened by using the same untargeted approach employed for the RPC metabolomics analysis. The resulting samples (s1–s18) were designed to characterize the impact of each enzyme and their combinations on the aforementioned compounds. BG-treated samples (s1, s7, s10, s13, and s16) were treated at 50 °C to investigate the enzyme’s ability to catalyze the cleavage of glycosidic bonds in the compounds. For example, samples s1 and s7 include sinapic acid alone and in combination with other compounds, while samples s10 and s13 focus on the effects of BG on K3OSS and K3OSS7OG, respectively. Sample s16 serves as a control to assess the baseline effect of the BG treatment. LAC-treated samples (s2, s8, s11, s14, and s17) underwent treatment with LAC at RT to investigate the enzyme’s influence on compound oxidation. Sample s2 included a mixture of sinapic acid, K3OSS, and K3OSS7OG, while samples s8, s11, and s14 examined the effect of LAC on individual compounds. Sample s17 acted as a control for the LAC activity. Combined LAC and BG (LB) samples (s3, s9, s12, s15, and s18) were treated with both enzymes in equal doses to evaluate their combined impact. Samples s3, s9, s12, and s15 were used to study the effect of this combined enzymatic action on sinapic acid, K3OSS, K3OSS7OG, and their mixture, respectively. Sample s18 was a control to assess the combined treatment’s baseline impact.

**Figure 5:**
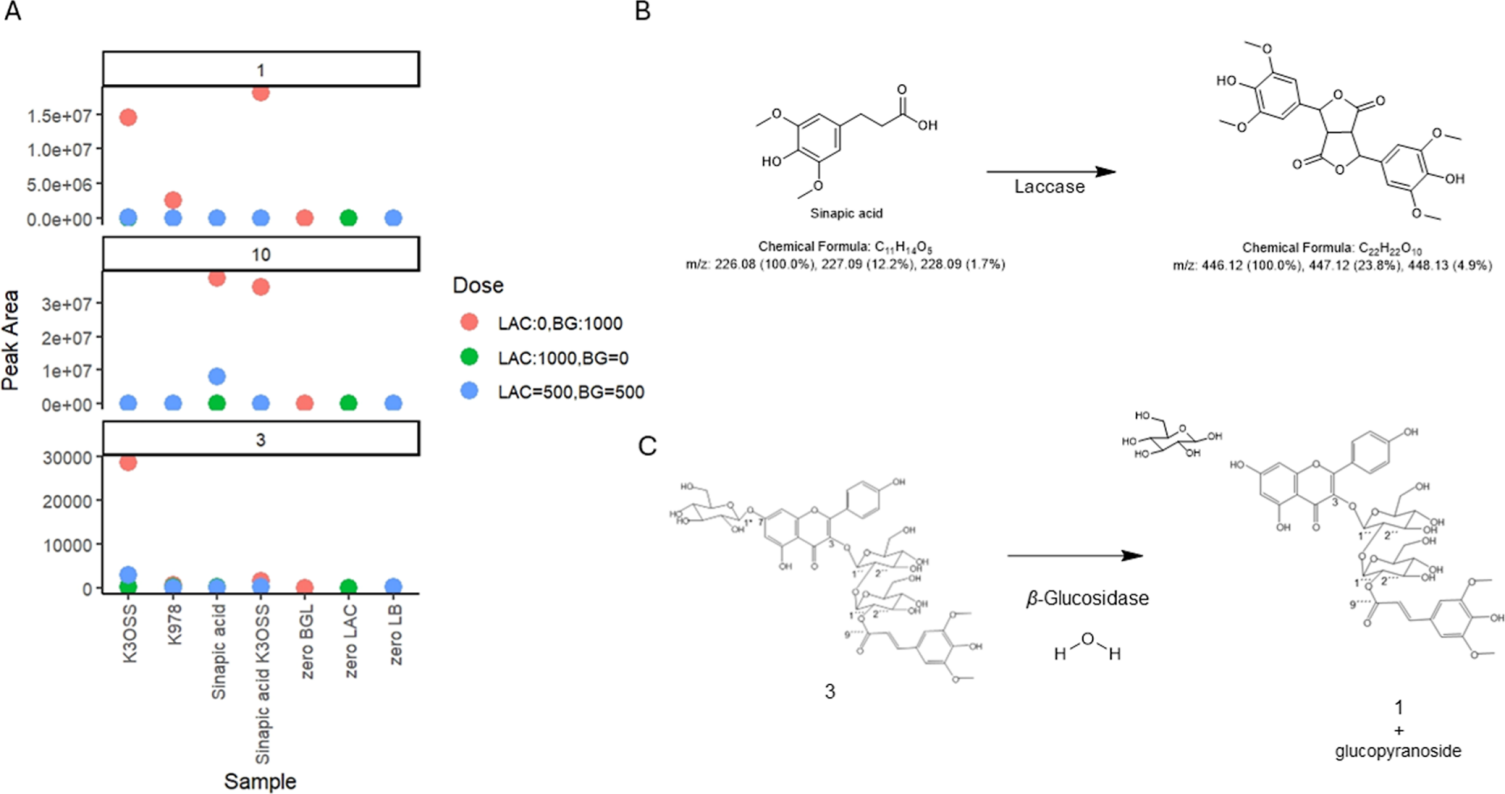
Multipanel graphical summary of the single reference compound treatment. (A) Dose–response scatter plot displaying peak areas of compounds **1** (K3OSS), **3** (K3OSS7OG), and **10** (sinapic acid) in various samples. The *Y*-axes represent the peak areas as a measure of intensity, and the *X*-axes categorize the samples spiked with different compounds and enzyme doses. The samples include controls and those treated with K3OSS7OG, sinapic acid, K30SS, and a mixture of the three. Dose groups are color-coded for clarity: LAC:0, BG:1000 in green, LAC:1000, BG:0 in red, and LAC:500, BG:500 in blue. (B,C) Proposed resulting reactions of the BG and LAC enzymes’ treatment.

Figure 5A summarizes the results from the UHPLC-qTOF-MS analysis of the samples in this experiment. Compound **1** was detected in both s1 and s10, confirming that BG does not catalyze cleavage of the glycosidic bond in this metabolite. Additionally, the presence of compound **1** in sample s13 indicates that when compound **3** is treated with BG, it is degraded into compound 1 (Figure 5C). These findings align with the hypothesis formulated from the metabolomic screening of the RPC samples.

When combined with the sensory data shown in Figure 2, these results further support existing sensomics literature on the taste-active compounds in rapeseed protein products.^6^ They also explain the observed increase in the average perceived bitterness (Figure 2). Sinapic acid was found in the BG-treated samples (s1 and s7) and not in the LAC-treated samples (s2 and s8; Figure 5A), which indicates that treatment with LAC catalyzes the degradation of this metabolite, while BG treatment does not result in degradation. Compound **3** was detected in sample s13, although at a lower intensity than in the untreated control. This result suggests that the compound was not completely degraded with BG, which could also be observed in RPC samples treated with BG. The treatment of this compound with LAC was effective in reducing its intensity.

To clarify the effect of LAC, features generated during the treatment were searched in the MSDIAL’s alignment file. A feature with *m*/z 445.12 eluting at 8.7 min with MS/MS fragments at *m*/*z* 342.11, 219.07, and 139.04 was selected as one of the most intense features in sinapic acid-containing samples s8 and s9, both of which underwent LAC-catalyzed oxidation. This monoisotopic mass suggests the presence of a molecule with the molecular formula C_22_H_22_O_10_. This mass has been reported in the literature to be related to the dimeric oxidation product of sinapic acid with LAC, suggesting that the electron supply, proton transfer, and the intermolecule C–C coupling reaction took place.^15^ Interestingly, according to the literature, the dimeric product resulting from this reaction is suggested to be 2,6-dimethoxy-*p*-benzoquinone (dehydrogenase acid dilactone; Figure 5B).^26^ In addition, another prominent feature obtained during the treatment of sinapic acid with LAC corresponds to *m*/*z* 891.23 eluting at 8.67 min with MS/MS fragments at *m*/*z* 423.09, 341.09, and 189.05. This *m*/*z* ratio suggests that the tetramer is also formed, further supporting the polymerization hypothesis even though the literature has not hypothesized this feature’s structure. These products could be found in the single reference compounds treated with LAC but could not be found in the RPC-treated samples LAC1, LB1, and LB2. This is most likely connected to the more complex set of oxidation and polymerization reactions taking place in the RPC system compared to that in the simple model system. Another study investigated the effect of the oxidation of **10** catalyzed by peroxidase and hydrogen peroxide.^27^ In addition to the previously mentioned dilactone product, the authors of this work identified five other products including a tetramerized product. According to the reaction products identified in this paper, the acquired UHPLC-qTOF–MS data were screened for monoisotopically deprotonated *m*/*z*: 418.13 (C_21_H_22_O_9_), *m*/*z*: 444.11 (C_22_H_20_O_10_), and *m*/*z*: 830.24 (C_43_H_46_O_17_), but no positive hits were found.

Samples containing compounds **1** and **3** treated with LAC did not appear to have features in the UHPLC-qTOF-MS-obtained alignment file. This lack of signals might be due to the polymerization of the larger kaempferols (compared to sinapic acid), which results in oxidation products that exceed our Dalton range of analysis (50–1500). However, the polymerization effect is hypothesized to have occurred due to the darker/brown color of the LAC-treated rapeseed protein (observational data). It is established in the literature that polyphenol oxidation via LAC treatment leads to polymerization and browning.^13,28^

Polyphenol oxidases (PPOs) generally act as either tyrosinase or catechol oxidase. The observed results indicate that a polymerization process occurs in this context, as browning was observed in all LAC-treated samples, except for LAC2 and LAC3, which served as untreated controls. In addition to the effect on compound **10**, PPO also oxidizes monophenols, such as the hydroxyl group at position 7 of compound **1** and the hydroxyl group of sinapic acid (**10**). This process involves the conversion of monophenols to diphenols, followed by the subsequent oxidation of *o*-diphenols to *o*-quinones.^28^ Fungal cresolases are known to catalyze the oxidation of monophenols to diphenols, which can then be further transformed into *o*-quinones, as observed in our experiments.^28^ Regarding the structures affected by LAC treatment shown in Figure 1, none possess *o*-diphenol groups. This suggests that tyrosinase activity may be occurring. Upon the formation of diphenols, *o*-quinones can be produced, leading to oxidative coupling, polymerization, and, ultimately, enzymatic browning.^28^ The presence of amino acids, especially nucleophilic amino acids, in rapeseed protein isolates might also play a role as an intermediate reaction in PPO-catalyzed polyphenol oxidation.^29^

## Conclusions

This study investigated the potential of enzymatic treatments to enhance the sensory profile of rapeseed protein. LAC treatment was particularly effective in reducing the levels of kaempferol derivatives containing a sinapic acid moiety, especially the bitter compound **1**, resulting in a notable decrease in bitter taste. In contrast, treatment with BG alone led to the breakdown of precursor metabolites, increasing the levels of **1** and, consequently, the perceived bitterness of the product.

The use of untargeted SWATH-based metabolomics to analyze the RPC samples and the pure reference compounds post-LAC and BG provided a molecular understanding of the enzymes’ impact on the rapeseed protein metabolome.

Future research directions may include the search for a more specific BG that can selectively cleave the glucose at position C(3) and the development of a deeper understanding of the degradation processes occurring during incubation of RPC in water at RT.

Overall, these results strongly support the potential of LAC-mediated enzymatic modification as an effective biotechnological strategy to enhance the sensory quality of rapeseed protein products suitable for rapeseed-protein-containing food and beverage products with superior taste profiles.

## Supporting Information Available

Larger version of the untargeted metabolomics heatmap including the features’ names; bar plots of the peak areas of features 1183.3158_6.855, 977.25604_5.282, 977.25691_6.305, 1183.31659_6.699, and 815.2074_6.694 across different samples; and table reporting the concentrations of kaempferol in various samples subjected to different enzyme treatments (PDF)
