# Supplementary material for: Reducing the Bitterness of Rapeseed Protein: Integrating Enzymatic Treatment, Metabolomics, and Sensory Analysis to Elucidate Underlying Mechanisms
Source: J Agric Food Chem. 2025 Feb 2;73(6):3657–68. doi: 10.1021/acs.jafc.4c10442 (PMC11826997; doi:10.1021/acs.jafc.4c10442)
Supplement: Supplementary file 1 — jf4c10442_si_001.pdf [file jf4c10442_si_001.pdf]

Supplementary materials

Reducing the Bitterness of Rapeseed Protein: Integrating Enzymatic Treatment, Metabolomics, and Sensory Analysis to Elucidate Underlying Mechanisms

Andrea Spaccasassi [†*&], Christoph Walser [†&], Anni Nisov [$], Nesli Sozer [$], Oliver Frank[†], Corinna Dawid [†*^] and Thomas F. Hofmann [†]

[†]Chair of Food Chemistry and Molecular and Sensory Science, Technical University of Munich, Lise-Meitner-Str. 34, D-85354 Freising, Germany,

[*] TUM CREATE, 1 CREATE Way, #10-02 CREATE Tower, Singapore, 138602 Singapore

[$] VTT Technical Research Centre of Finland Ltd., Tietotie 2, 02044 VTT Espoo, Finland.

[^] Professorship for Functional Phytometabolomics, TUM School of Life Sciences, Technical University of Munich, Lise-Meitner-Str. 34, 85354 Freising, Germany

[&] These authors contributed equally.

---

[*] **To whom correspondence should be addressed**

PHONE +49-8161-712902

FAX +49-8161-712949

E-MAIL thomas.hofmann@tum.de; corinna.dawid@tum.de

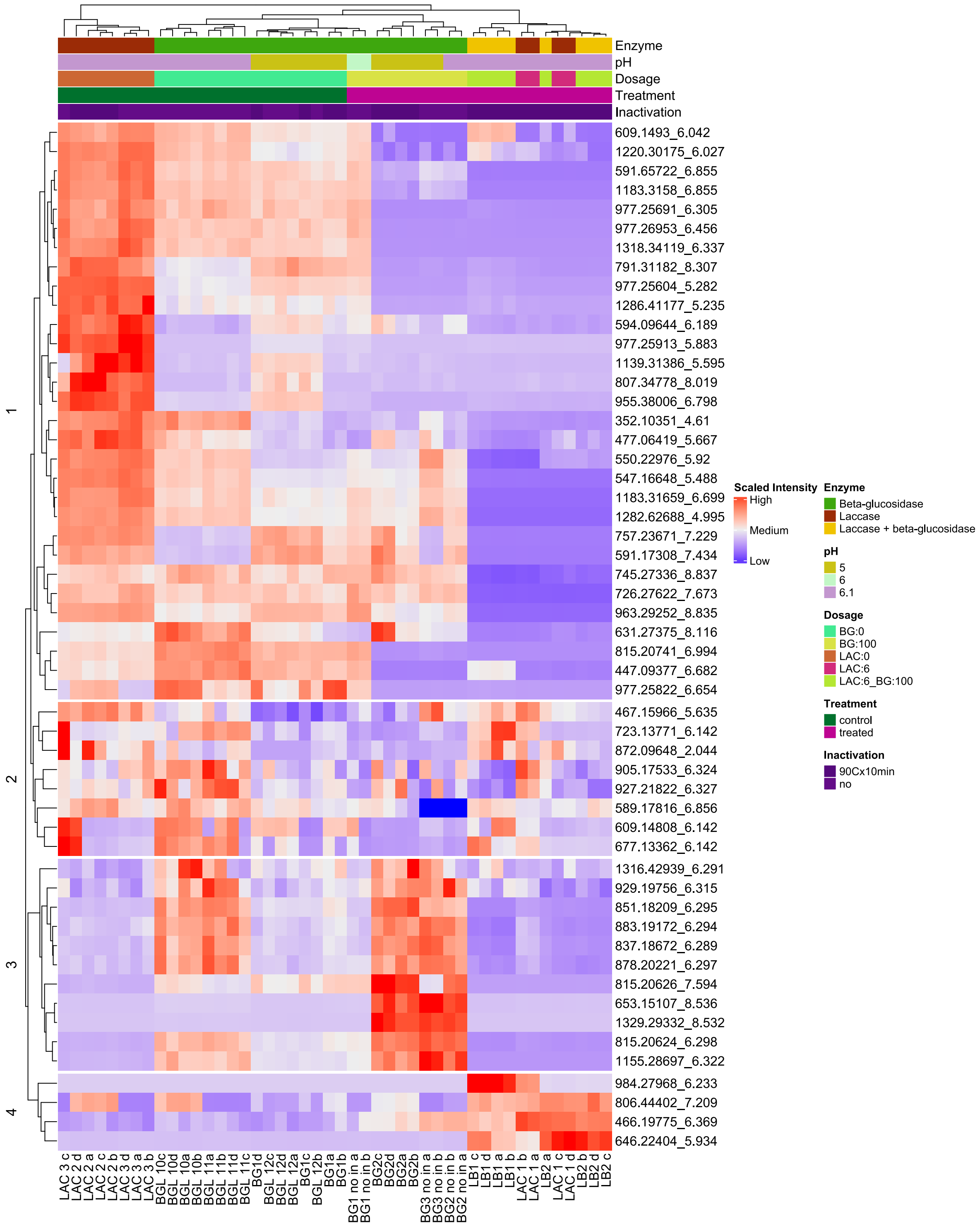

Figure S1

Larger version of the untargeted metabolomics heatmap including the features's names (Figure 3C main manuscript). This heatmap reports the intensity distribution of the selected feature from the alignment file, where target fragment masses were identified. Color is based on the scaled peak area extracted from the peak table.

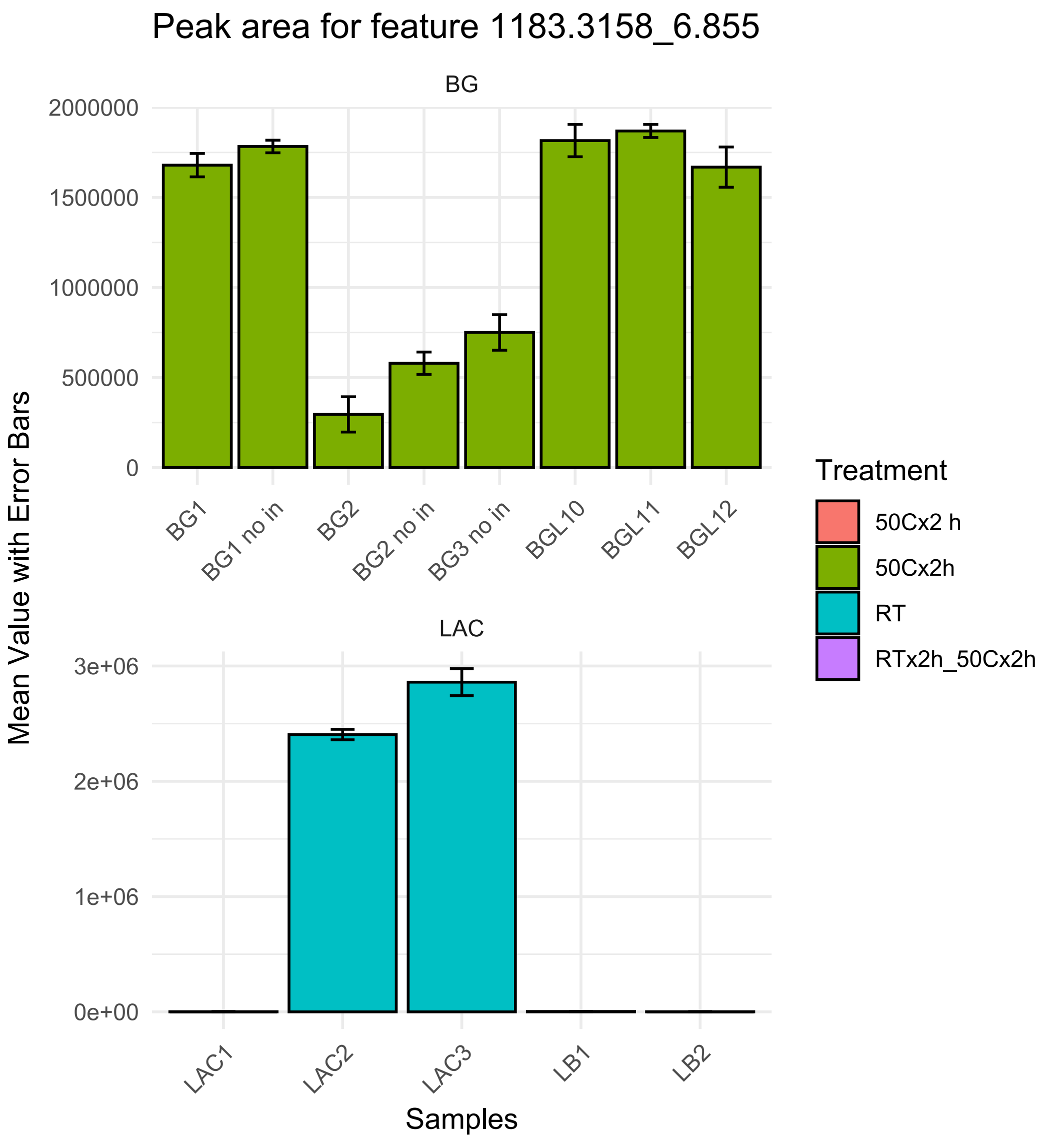

Figure S2

Mean peak area of feature 1183.3158_6.855 across different samples. The bars represent the mean values, while the error bars indicate the standard deviation for each sample within the respective treatment groups. Samples are grouped by the enzyme treatment and presented in different facets to allow for independent y-scales.

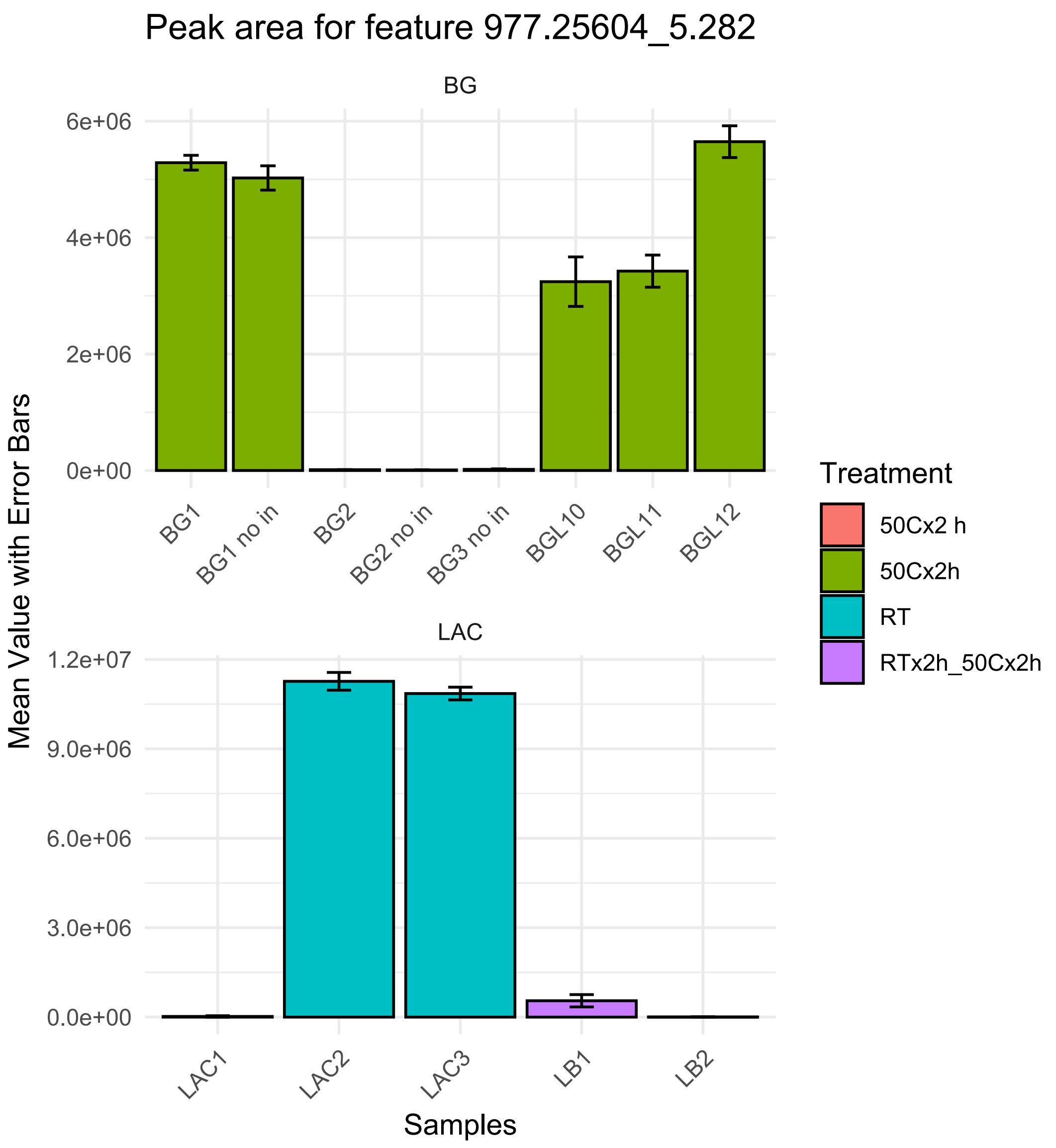

Figure S3

Mean peak area of feature 977.25604_5.282 across different samples. The bars represent the mean values, while the error bars indicate the standard deviation for each sample within the respective treatment groups. Samples are grouped by the enzyme treatment and presented in different facets to allow for independent y-scales.

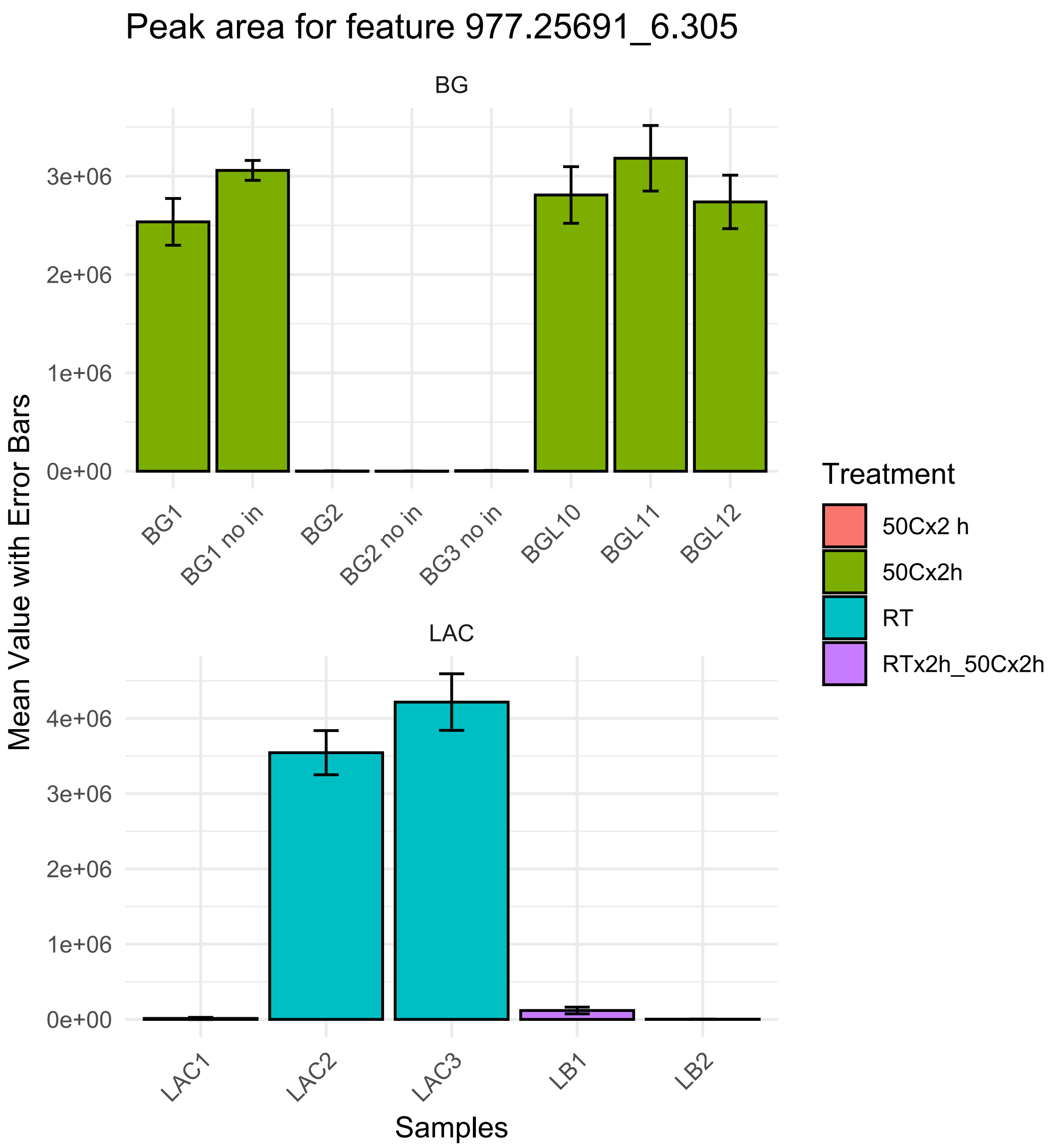

Figure S4

Mean peak area of feature 977.25691_6.305 across different samples. The bars represent the mean values, while the error bars indicate the standard deviation for each sample within the respective treatment groups. Samples are grouped by the enzyme treatment and presented in different facets to allow for independent y-scales.

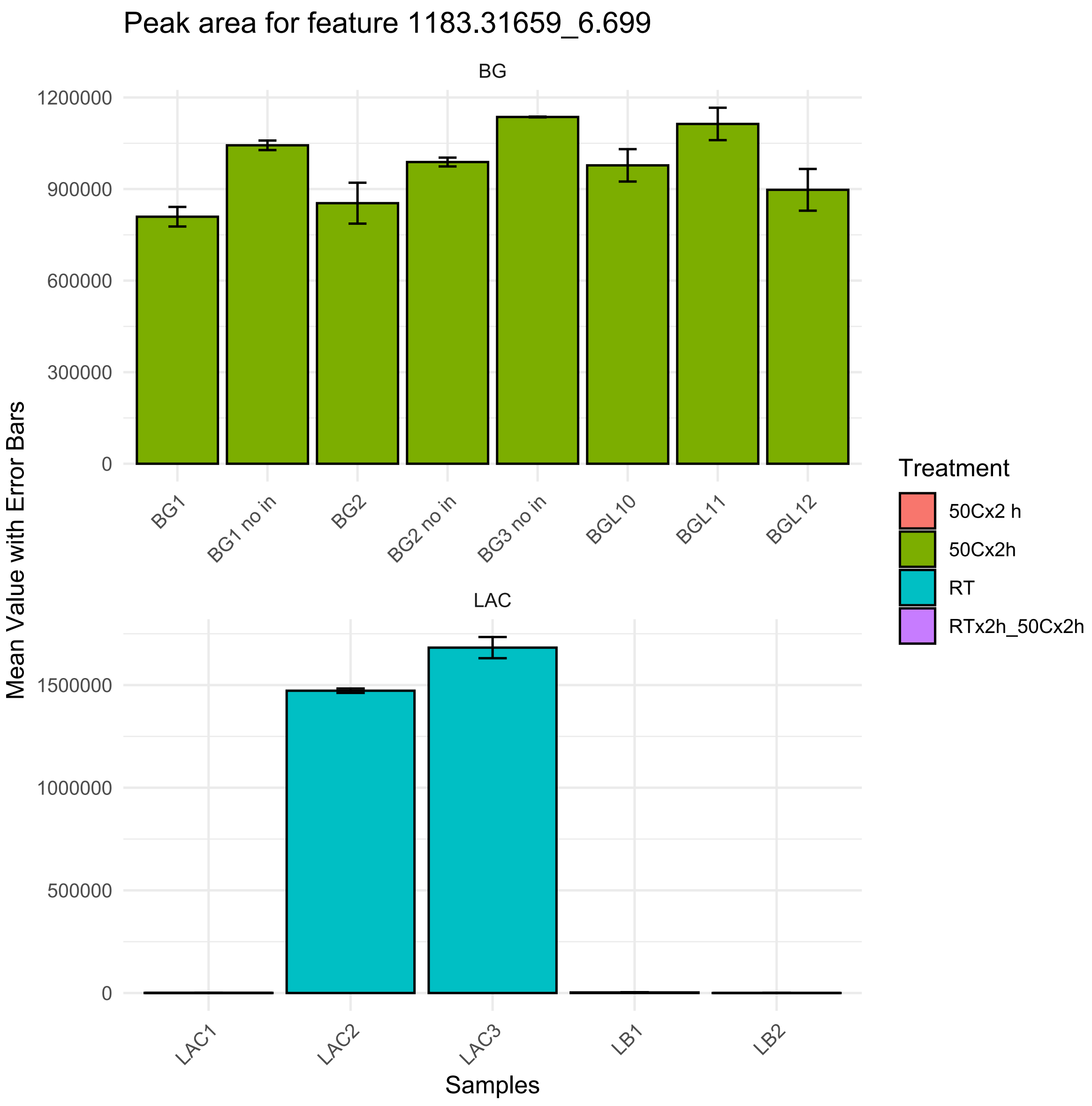

Figure S5

Mean peak area of feature 1183.31659_6.699 across different samples. The bars represent the mean values, while the error bars indicate the standard deviation for each sample within the respective treatment groups. Samples are grouped by the enzyme treatment and presented in different facets to allow for independent y-scales.

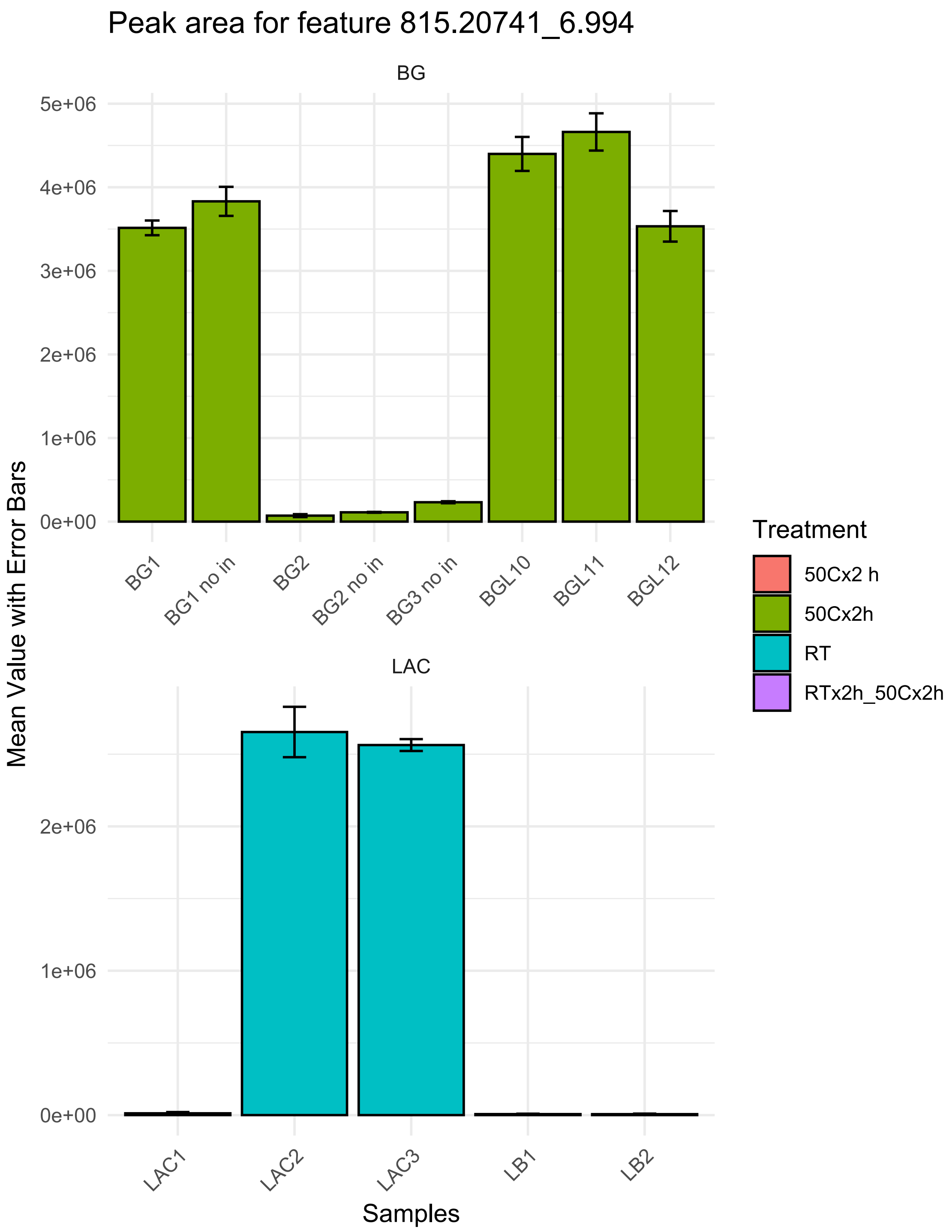

Figure S6

Mean peak area of feature 815.2074_6.694 across different samples. The bars represent the mean values, while the error bars indicate the standard deviation for each sample within the respective treatment groups. Samples are grouped by the enzyme treatment and presented in different facets to allow for independent y-scales.

Table S1

Concentrations of kaempferol in various samples subjected to different enzyme treatments. The table lists the sample codes, corresponding enzyme treatments (BG for β-glucosidase, LAC for laccase, LB for a combination of both enzymes), and the resulting concentrations of kaempferol (mg/g) measured in each sample.

Samples	Kampferol	Concentration mg/g
BG1	2	0.012
BG2	2	0.000
BG3	2	0.000
BGL10	2	0.007
BGL11	2	0.006
BGL12	2	0.013
LAC1	2	0.004
LAC2	2	0.127
LAC3	2	0.128
LB1	2	0.083
LB2	2	0.001
BGL1 no in	2	0.008
BGL2 no in	2	0.000
BGL3 no in	2	0.001
BG1	3	0.613
BG2	3	0.002
BG3	3	0.001
BGL10	3	0.403
BGL11	3	0.411
BGL12	3	0.630
LAC1	3	0.002
LAC2	3	0.823
LAC3	3	0.808
LB1	3	0.066
LB2	3	0.000
BGL1 no in	3	0.564
BGL2 no in	3	0.000
BGL3 no in	3	0.003
BG1	4	0.006
BG2	4	0.000
BG3	4	0.000
BGL10	4	0.000
BGL11	4	0.000
BGL12	4	0.010
LAC1	4	0.000
LAC2	4	0.030
LAC3	4	0.032

LB1	4	0.000
LB2	4	0.000
BGL1 no in	4	0.003
BGL2 no in	4	0.000
BGL3 no in	4	0.000
BG1	5	2.425
BG2	5	0.013
BG3	5	0.015
BGL10	5	2.370
BGL11	5	2.405
BGL12	5	2.443
LAC1	5	0.027
LAC2	5	2.431
LAC3	5	2.410
LB1	5	2.254
LB2	5	0.048
BGL1 no in	5	2.410
BGL2 no in	5	0.008
BGL3 no in	5	0.019
BG1	1	0.182
BG2	1	0.364
BG3	1	0.372
BGL10	1	0.291
BGL11	1	0.288
BGL12	1	0.171
LAC1	1	0.018
LAC2	1	0.072
LAC3	1	0.057
LB1	1	0.004
LB2	1	0.019
BGL1 no in	1	0.216
BGL2 no in	1	0.391
BGL3 no in	1	0.388
BG1	6	0.499
BG2	6	0.000
BG3	6	0.000
BGL10	6	0.503
BGL11	6	0.521
BGL12	6	0.508
LAC1	6	0.000
LAC2	6	0.536
LAC3	6	0.527
LB1	6	0.005
LB2	6	0.000
BGL1 no in	6	0.535
BGL2 no in	6	0.000
BGL3 no in	6	0.001
BG1	8	0.789

BG2	8	0.004
BG3	8	0.004
BGL10	8	0.825
BGL11	8	0.863
BGL12	8	0.786
LAC1	8	0.006
LAC2	8	0.389
LAC3	8	0.381
LB1	8	0.340
LB2	8	0.018
BGL1 no in	8	0.736
BGL2 no in	8	0.002
BGL3 no in	8	0.005
BG1	7	0.015
BG2	7	0.004
BG3	7	0.004
BGL10	7	0.015
BGL11	7	0.015
BGL12	7	0.015
LAC1	7	0.000
LAC2	7	0.015
LAC3	7	0.015
LB1	7	0.000
LB2	7	0.000
BGL1 no in	7	0.014
BGL2 no in	7	0.004
BGL3 no in	7	0.005
BG1	9	0.077
BG2	9	0.002
BG3	9	0.003
BGL10	9	0.078
BGL11	9	0.079
BGL12	9	0.073
LAC1	9	0.000
LAC2	9	0.043
LAC3	9	0.036
LB1	9	0.000
LB2	9	0.000
BGL1 no in	9	0.072
BGL2 no in	9	0.003
BGL3 no in	9	0.005
